# Depletion of gut microbiota influents glucose metabolism and hyperandrogenism traits of mice with PCOS induced by letrozole

**DOI:** 10.3389/fendo.2023.1265152

**Published:** 2023-10-20

**Authors:** Yushan Li, Yuchen Zhu, Dan Li, Wen Liu, Yi Zhang, Wei Liu, Chenhong Zhang, Tao Tao

**Affiliations:** ^1^ Department of Endocrinology and Metabolism, Renji Hospital, School of Medicine, Shanghai Jiao Tong University, Shanghai, China; ^2^ State Key Laboratory of Microbial Metabolism, School of Life Sciences and Biotechnology, Shanghai Jiao Tong University, Shanghai, China

**Keywords:** polycystic ovary syndrome, gut microbiota, pseudo germ-free, glucose metabolism, hyperandrogenemia

## Abstract

**Background:**

Polycystic ovary syndrome (PCOS) is a multifaceted disorder that impacts metabolism, reproduction, as well as endocrine function, characterized by excessive levels of androgen and insulin resistance. The gut microbiota has been implicated in the pathogenesis of PCOS. However, the precise mechanisms through which the gut microbiota influences PCOS still require further elucidation.

**Methods:**

The PCOS mouse model was established through the administration of letrozole to both conventional and antibiotics-treated mice. The evaluation of glucose metabolism, sex hormone levels, and ovarian morphology was conducted. Furthermore, the fecal samples from each group of mice were subjected to 16S rRNA gene sequencing, and functional prediction of gut microbiota was proceeded using PICRUSt2 to explore potential mechanisms.

**Results:**

By using letrozole-induced PCOS mice model, we manifested that antibiotic intervention significantly reduced the serum total testosterone level and ameliorated glucose intolerance. Antibiotic treatment reduced the number of amplicon sequence variants (ASVs), as well as the Shannon and Simpson index. Meanwhile, letrozole induced a significant increase in the Shannon and Simpson index instead of ASVs. Through random forest model analysis, the results revealed significant alterations in three distinct groups of microbiota, namely Clostridia_vadinBB60_group, Enterorhabdus, and Muribaculaceae after letrozole treatment. Further correlation analysis revealed a positive association between alterations in these microbiota and both serum total testosterone levels and the area under the curve (AUC) of blood glucose in IPGTT. The administration of antibiotics led to a decrease in the absolute abundance of 5 ASVs belonging to unclassified Clostridia_vadinBB60_group, unclassified Enterorhabdus, and unclassified Muribaculaceae, which exhibited a positive correlation with the levels of total testosterone in mice serum, as well as the area under the curve of blood glucose in IPGTT. Moreover, 25 functional pathways of gut microbiome were significantly discrepant between the letrozole-treated mice with and without antibiotics.

**Conclusion:**

These results suggest that disturbance of the gut microbiota may take participate in the progression of PCOS and manipulating the composition of the gut microbiota may be a therapeutic approach for managing PCOS.

## 1 Introduction

Polycystic ovary syndrome (PCOS) is one of the most prevalent metabolic and reproductive disorder affecting a significant percentage of women in their reproductive years, ranging from 4% to 20% (1), and is primarily characterized by hyperandrogenism and ovarian dysfunction (2), which brings a heavy social and economic burden to patients (3). Women with PCOS also face a higher probability of enduring endocrine comorbidities and developing cardiometabolic diseases (4). The current body of research indicates that PCOS is a multifaceted polygenic disorder affected by a combination of genetic and environmental elements, such as diet and lifestyle (5). However, the pathogenesis of PCOS is still unrevealed.

The principal pathological foundation of PCOS is hyperandrogenism and insulin resistance. Studies have shown a strong correlation between the disruption of gut microbiome and the development of hyperandrogenism and insulin resistance (6–11). The gut microbiota and various substances it produces, including short-chain fatty acids, lipopolysaccharides, bile acids, and branched-chain amino acids, exert a significant impact on interorgan communication by modulating gut barrier function and influencing peripheral tissue physiology and metabolism, potentially contributing to the pathogenesis of obesity, insulin resistance and impaired ovarian function (12). In 2012, Tremellen (13) introduced the DOGMA theory, positing that inadequate dietary habits can disrupt the equilibrium of gut microbiota, contributing to heightened intestinal mucosal permeability, which in turn allows for the infiltration of lipopolysaccharide (LPS) from Gram-negative colon bacteria by entering the bloodstream, thus triggering the immune response, and impeding insulin receptor function, ultimately resulting in increased levels of insulin and androgens in individuals with PCOS. Mice treated with letrozole co-culturing with control mice showed significant improvements in both reproductive and metabolic PCOS phenotypes compared to letrozole-treated mice (14). Qi et al. (7) discovered an increased abundance of Bacteroides vulgatus (B. vulgatus) in the gastrointestinal microbiota of patients with PCOS, which displayed an inverse relationship with the quantities of glycodeoxycholic acid (GDCA) and tauro ursodeoxycholic acid (TUDCA). Furthermore, the findings of mechanism studies indicated that GDCA activates the GATA3 pathway of intestinal group 3 innate lymphoid cell (ILC3), thereby stimulating the secretion of IL-22 (7). This process subsequently promoted the browning of white fat and inhibits ovarian inflammation, ultimately leading to an improvement in the PCOS-like phenotype. The transplantation of intestinal microbiota from rats with PCOS induced by DHEA into pseudo-germfree recipients resulted in impairment of glucolipid metabolism in the liver and an imbalance of reproductive hormones (6).

The pathophysiology of PCOS remains incompletely understood, necessitating the use of animal models to investigate its disease mechanism *in vivo*. Currently, there is no universally accepted model that can fully replicate PCOS-related symptoms. Various rodent models manifesting PCOS-like traits can be induced through the administration of diverse drugs and szintetikus hormones, including androgens containing dihydrotestosterone (DHT), testosterone (T), and dehydroepiandrosterone (DHEA), estrogen, as well as aromatase inhibitors (15). Aromatase, which is encoded by Cyp19a1, is a cytochrome p450 enzyme essential for the conversion of androgens to estrogens (16). Letrozole, a type of aromatase inhibitor that is not derived from steroids, possesses the capability of reducing estrogen levels while simultaneously boosting the production of endogenous androgens, grounded on the discovery that genetic variations in Cyp19a1 are linked to PCOS in females, while also disrupting the LH/FSH ratio (17). Additionally, the follicular fluid obtained from the ovaries of women with PCOS demonstrates an elevated androgen to estrogen ratio, attributed to heightened 17α-hydroxylase activity (16). This mechanism helps in the prevention of ovulation in animal ovaries and mimics the neuroendocrine manifestations of PCOS. Letrozole-induced mice exhibit similar sex hormone levels, glucose metabolism, and ovarian morphology to those of PCOS patients (18, 19). Consequently, the utilization of letrozole-induced mice presents a viable approach for investigating pathophysiology of PCOS.

Furthermore, the utilization of a combination of antibiotics, comprising vancomycin hydrochloride, ampicillin sodium, neomycin sulfate, and metronidazole, has exhibited effectiveness in eliminating the indigenous gut microbiota of the host (20). These antibiotics are commonly employed alongside, or occasionally in lieu of, sterile mice to inspect the involvement of the gut microbiota in particular pathological conditions (21, 22). The alteration of the bile acid (BA) pool by antibiotic-induced microbiome-depleted (AIMD) can have an impact on metabolic homeostasis due to the capability of BAs to modulate host metabolism via activating the g protein-coupled BA receptor (TGR5) and farnesoid X receptor (FXR) signaling pathways (23–25). The administration of antibiotics to mice resulted in a noteworthy reduction in short-chain fatty acid (SCFA) levels, an increase in plasma baseline GLP-1 levels, and a rise in proglucagon (Gcg) expression in the colon (26).

Thus, our investigation endeavored to make model of a pseudo germ-free PCOS mouse through the application of letrozole and antibiotics, with the aim of allowing insight into the gut microbiota’s influence on the regulation of metabolic and reproductive phenotypes in mice with PCOS.

## 2 Methods

### 2.1 Animals

Forty female C57BL/6J mice aged 3 weeks were acquired from Shanghai Chengqin Laboratory Animal Co Ltd. In a vivarium designed to maintain specific pathogen-free (SPF) conditions, five mice were accommodated per cage. The vivarium was equipped with an automatic lighting system that provided a 12-hour light and 12-hour dark cycle, with the light period lasting from 06:00 to 18:00. The mice had unrestricted access to water and food. Weekly measurements were taken to determine the body weights of mice.

### 2.2 Induction of pseudo germ-free PCOS mouse model

A total of forty mice were stochasticly allocated into four separate groups (each containing ten mice): control, letrozole (LET), antibiotics (ABX), and antibiotics plus letrozole (ABX_LET). To induce the PCOS phenotype (19), mice in LET and ABX_LET groups were orally administered with 1 mg/kg of letrozole (Jiangsu Hengrui Pharmaceuticals Co., Ltd., China) dissolved in 0.5% Carboxy Methyl Cellulose (CMC) (Sigma, USA) over a 21-day period, once daily. Control and ABX groups received vehicle only (0.5% CMC). ABX and ABX_LET groups were treated with an antibiotic cocktail composed of vancomycin hydrochloride(0.5g/L), ampicillin sodium(1g/L), neomycin sulfate(1g/L), and metronidazole(0.2g/L) (20) daily in their drinkable water, while there was free access to water in the control and LET groups. And one mouse from the ABX group died.

### 2.3 Intraperitoneal Glucose Tolerance Test

Mice were subjected to a 12-hour fasting period prior to undergoing the IPGTT procedure. Blood glucose levels were determined by extracting blood samples from tail vein with a blood glucose meter (Accu-Chek Performa; Roche, USA). After measuring the fasting glucose levels, glucose solution was injected intraperitoneally in mice at a dosage of 2 g/kg body weight for IPGTT, and tail blood samples were collected at intervals of 15, 30, 60, 90, and 120 minutes after glucose administration to measure glucose level. The fasting blood samples were gathered into tubes for the purpose of measuring insulin levels.

### 2.4 Analysis of serum

After completing the treatments, blood samples were gathered and subjected to centrifugation at a speed of 2500 rpm at 4°C for a duration of 30 min. Afterwards, the liquid above was meticulously transferred to cryogenic vials and promptly preserved at -80°C for subsequent examination. According to the manufacturer’s instructions, the levels of testosterone (CEA458Ge, Cloud-Clone Corp., China), luteinizing hormone (LH) (CEA441Mu, Cloud-Clone Corp., China), and insulin (80-INSMSU-E01, ALPCO, USA) underwent detection through utilizing enzyme-linked immunosorbent assay (ELISA) kits.

### 2.5 Ovary morphology check

Dissected ovaries were fixed in 4% paraformaldehyde, hydrated in gradient ethanol, and embedded in paraffin at the end of the experiment. The samples then underwent slide processing in preparation for H&E staining, with the purpose of analyzing any pathological structural changes. The cystic follicles (CF) and corpus luteum (CL) numbers were counted.

### 2.6 Sampling of feces, isolation of DNA, and sequencing of 16S rRNA genes

Prior to sacrifice, the fecal samples were collected and preserved at -80°C immediately till following work. DNA was extracted from stool samples using a method described earlier (27). In accordance with the manufacturer’s instructions, the V3-V4 regions sequencing library of 16S rRNA gene was built(part no.15044223 Rev. B; Illumina Inc., CA, USA) based on the previous publication with some modifications (28).

### 2.7 Detection of total fecal bacterial loads by qPCR

The plasmid from the Ruminococcus strain, which contained the full-length 16S gene at a concentration of 10^9^ copies/ml was diluted at different gradients to obtain concentrations of 10^8^, 10^7^, 10^6^, 10^5^, 10^4^, 10^3^, and 10^2^ copies/ml. The qPCR was conducted on a LightCycler 96 system using a 20μL reaction mixture comprising template (20 ng), primer Uni331F (5’-TCCTACGGGAGGCAGCAGT-3’), primer Uni797R (5’-GGACTACCAGGGTATCTAATCCTGTT-3’), and supermix (Bio-Rad), with Standard and sample DNA replicated twice (29, 30). A PCR reaction was performed using the following conditions: 95°C for 5 minutes, followed by 39 cycles of 95°C for 20 seconds, 60°C for 60 seconds, 95°C for 10 seconds, 65°C for 1 minutes, 97°C for 1 seconds, and 37°C for 30 seconds. A standard curve was generated by performing a linear fit of the copy number and CT value of the plasmid across various gradients. The DNA samples’ copy number was determined through a standard curve by utilizing Opticon Monitor 3.1. And the resulting conversion was copies per gram of wet feces.

### 2.8 Analysis of the 16S rRNA gene sequence

Quantitative Insights Into Microbial Ecology2 (QIIME2, v2018.11) (31) was applied for analysis of raw sequences. The “Demux” and “cutadapt” plugins were used for sequence quality and primer removal, respectively. Meanwhile, DADA2 (32) was used to obtain mass amplicon sequence variants (ASVs) by filtering, denoising, and merging (33). The construction of a phylogenetic tree for each ASV was achieved by utilizing “FastTree”, while the taxonomic classification was assigned through the SILVA132 16S rRNA database (34). The absolute abundance matrix of ASVs was obtained by multiplying the original abundance matrix by the number of bacteria (copies/g). ASVs number, Shannon index, and Simpson index were employed to assess the Alpha diversity of samples. Based upon the Principle Coordinate Analysis (PCoA) of Bray Curtis distance, Beta diversity was evaluated. Permutational multivariate analysis of variance test (PERMANOVA; 9,999 permutations) was employed to determine the significance of differences in intestinal microbiota between groups. Differences were defined significant when *P* was <0.05.

Using the “random Forest” package in R (v 4.2.0, USA), the method of random Forest and cross validation is utilized to find the ASVs that can distinguish different groups. Use the “pheatmap” package in R to visualize the absolute abundance of different ASVs across various groups. Spearman analysis was conducted on the correlation between ASV abundance and total testosterone and glucose metabolism by Origin (MicroCal, USA). When *P* was <0.05, the correlation was considered significant.

### 2.9 PICRUSt2 functional prediction analysis

PICRUSt2 (35) was used to build the prediction spectrum of gene function in the bacterial domain. And STAMP (36) was applied to analyze the PICRUSt2 prediction results, acquire metabolic pathway information with significant differences, and generate a visual representation of the findings. Clustering and correlation analysis and calculations were proceeded through the “stats” package in R.

### 2.10 Statistical analysis

The statistical significance of physiological and biochemical data among the different groups was assessed using one-way analysis of variance (ANOVA) with SPSS 22.0 software (SPSS Inc., Chicago, IL, USA) and GraphPad Prism 8.0 (GraphPad Software, Inc., San Diego, CA). The data conforming to a normal distribution was analyzed using the One-way ANOVA method. The K-S test was applied for statistical analysis of the data disconfirming to normal distribution. In order to compare the differences between the four groups, the P values were corrected through the Tukey post-test. A two-tailed unpaired student t test was employed to assess the differences between the two groups. Statistical significance was considered when the P value was equal to or less than 0.05.

## 3 Results

### 3.1 Androgen levels were decreased after antibiotics treatment of letrozole model mice but the polycystic ovary structure did not change

**Figure 1:**
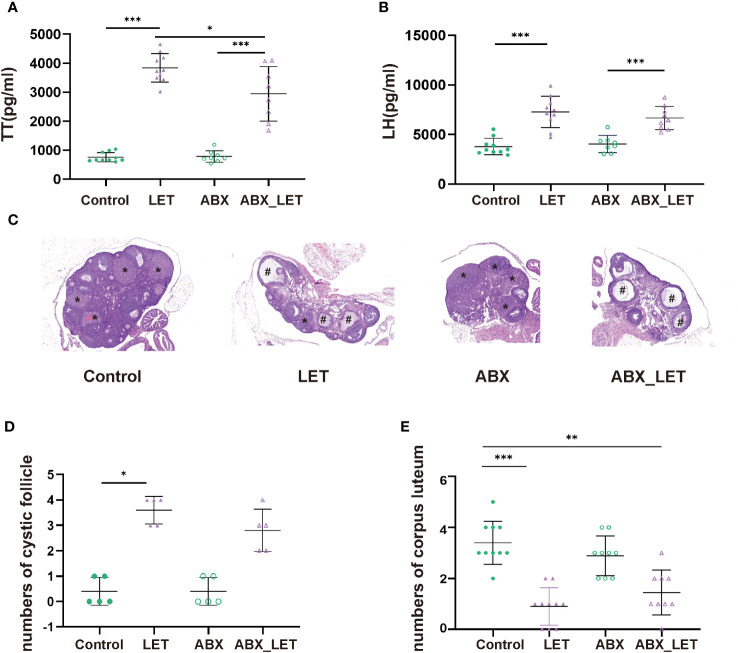
Serum sex hormone levels and ovary morphology of Letrozole-induced and pseudo germ-free mice **(A)** The level of total testosterone; **(B)** The level of luteinizing hormone. Data were presented as mean ± SD and analyzed using ANOVA; *P< 0.05, ***: P< 0.001. **(C)** Ovary morphology; **(D)** Numbers of corpus luteum; **(E)** Numbers of cyst-like follicles.

Letrozole was used by intragastric administration (at a concentration of 1 mg/kg diluted in a solution of 0.5% CMC.) to induce PCOS in C57BL/6 mice in letrozole (LET) and antibiotics plus letrozole (ABX_LET). Mice from antibiotics (ABX) and antibiotics plus letrozole (ABX_LET) groups were treated with an antibiotic cocktail, while the control and LET groups received distilled water. At the conclusion of the treatments, blood samples were acquired and the concentrations of testosterone, luteinizing hormone (LH) and insulin were tested. Hyperandrogenism is currently considered to be one of the most essential characteristics of PCOS, while elevated luteinizing hormone (LH) level is considered to be one of the features of neuroendocrine disorders of PCOS (37). Therefore, fasting serum sex hormone levels of four groups of mice were detected respectively. The LET group demonstrated a significant increase in both total testosterone (TT) and LH levels when compared to the Control group, and the ABX_LET group exhibited significantly lower TT levels compared to the LET group (**Figures 1A, B**). But antibiotics treatment did not affect LH levels in ABX_LET group (**Figure 1B**). It is noteworthy that the TT and LH levels in ABX_LET group were significantly elevated compared to the ABX group (**Figures 1A, B**), suggesting additional factors influencing the androgen levels in PCOS mice. The changes in ovarian structure were determined by observing the ovarian tissue sections of mice. The morphological structure of the ovarian tissues of Control and ABX group was generally normal, with no cystic follicles and multiple luteal bodies (**Figure 1C**). Compared with Control group, the ovarian tissue in the LET group displayed aberrant overall structure, characterized by polycystic changes, a significant increase in number of cystic follicles, a decrease in number of luteum present in the ovary (**Figures 1C–E**). Compared with LET group, the ovarian structure of mice in ABX_LET group was not significantly improved, and no significant reduction of cystic follicles was observed (**Figures 1C–E**). The findings validated the successful induction of hyperandrogenemia and neuroendocrine symptoms in mice with PCOS, and suggested that the gut microbiome may participate in the onset of hyperandrogenism instead of LH level and ovarian structure.

### 3.2 Glucose metabolism was improved after antibiotics treatment in letrozole-induced PCOS mice

**Figure 2:**
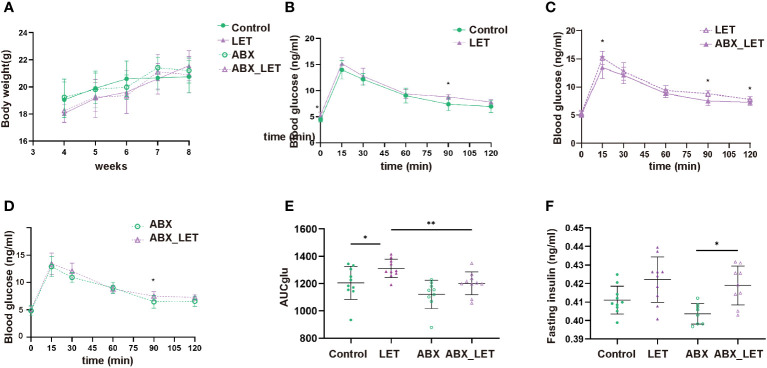
Weight, blood glucose test, and insulin level of Letrozole-induced and pseudo germ-free mice **(A)** weight; **(B–D)** Curves of blood glucose levels during Intraperitoneal Glucose Tolerance Test (IPGTT); **(E)** IPGTT areas under the curve (AUC); **(F) **Fasting serum insulin. Data were presented as mean ± SD and analyzed using ANOVA; *:P< 0.05, **:P< 0.01.

After modeling by letrozole for 21 consecutive days, the glucose metabolism of mice in the four groups was evaluated by IPGTT, body weight, and fasting insulin level. The four groups showed no significant differences in body weight. (**Figure 2A**). The fasting blood glucose levels, 90-minute postprandial blood glucose levels, and the area under the curve during IPGTT exhibited significantly higher values in the LET group compared to the Control group (**Figures 2B, E**). The ABX_LET group displayed lower levels of blood glucose at 15, 90, and 120 min and area under the IPGTT curve than the LET group (**Figures 2C, E**) (all P<0.05). The result above demonstrated that the mice belonging to the LET group exhibited glucose tolerance impairment and antibiotic treatment effectively alleviated glucose tolerance. However, the comparison of fasting insulin levels between four groups showed that no significant difference was found between the Control group and the LET group, nor between LET and ABX_LET group (**Figure 2F**), indicating that gut microbiota in PCOS mice may not affect their fasting insulin level. Interestingly, it was also found that compared to ABX group, the blood glucose level of mice in ABX_LET group increased significantly at 90 min and the area under the IPGTT curve showed a significant difference (**Figures 2D, E**), and a significant increase in fasting insulin level was observed in ABX_LET group compared to ABX group(**Figure 2F**), which suggested other factors that affected the glucose metabolism of PCOS mice.

### 3.3 Letrozole and antibiotics treatment caused alterations in the composition of gut microbiota of mice

**Figure 3:**
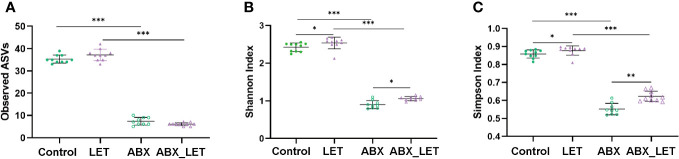
The diversity of gut microbiota in Letrozole-induced and pseudo germ-free mice **(A)** Observed ASVs; **(B)** Shannon index; **(C)** Simpson Index. Data are presented as the mean ± SD and analyzed using the ANOVA test. *P< 0.05, **P< 0.01, **P<0.001, ***P<0.001.

With an aim of exploring the effects of antibiotics on the diversity of gut microbiota of letrozole-induced PCOS mice, sequencing was conducted on the 16S rRNA gene V3-V4 region of stool specimens acquired. The findings of the study suggested that the administration of antibiotics resulted in a markedly decrease in the quantity of amplicon sequence variants (ASVs), as well as the Shannon and Simpson indices of the intestinal flora in comparison to the two groups that did not receive antibiotic treatment (**Figures 3A–C**). Moreover, the LET group exhibited a remarkable elevation in the Shannon and Simpson indices, in comparison to the two groups without letrozole induction (**Figures 3A–C**). These results indicated that combined antibiotic intervention to gut microbiota could alter the diversity of intestinal flora in letrozole-induced mice.

**Figure 4:**
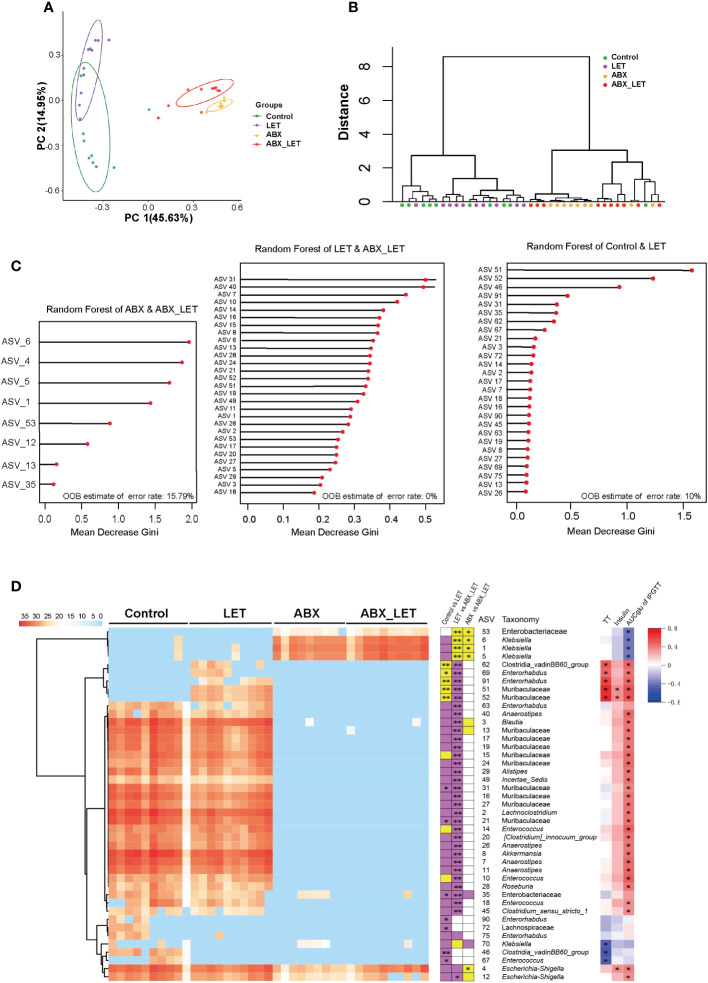
The structure of gut microbiota in Letrozole-induced and pseudo germ-free mice **(A)** PCoA plot of gut microbiota based on the Bray-Curtis distance. **(B)** clustering of the gut microbiota between different groups calculated with the PerMANOVA test using Bray-Curtis distances. *P<0.05, **P< 0.01.**(C)** ASVs to distinguish between the Control group and LET group. ASVs to distinguish between the LET group and ABX_LET group. ASVs to distinguish between the ABX group and ABX_LET group. **(D)** The members of the gut microbiota responding to Letrozole-induced and pseudo germ-free based on the random forest model. Left, the heatmap represents the normalized and log2 transformed relative abundance of the 43 ASVs in each sample; These ASVs were clustered by the ward. D method. Medium, the stacked bar plot indicates the direction in which the absolute abundance of ASVs varies between groups; Purple blocks indicate if the absolute abundance of 43 ASVs is more than the previous group, while yellow refers to less. The abundance of ASVs follows the same rule compared to the latter groups; The absolute abundance of key ASVs was compared between groups by Mann Whitney U test, * P < 0.05, **P < 0.01. Right: Absolute abundance of ASVs and Spearman correlation analysis of total testosterone, fasting insulin, and area under the IPGTT curve. Red was positively correlated and blue was negatively correlated, *P<0.05.

Beta diversity is frequently used to indicate the resemblance of the constituent part of intestinal flora across groups. To this end, the absolute abundance matrix of ASV was utilized to compute the Bray Curtis distance, followed by the execution of Principal coordinate analysis (PCoA) and PerMANOVA analyses on the basis of this distance metric. The findings revealed that the intestinal flora of the Control and LET groups exhibited significant distinctions from the ABX_LET group along the first principal coordinate (PC1) axis, which accounted for 45.63% of the total microbiota variance. The clustering analysis revealed that the flora structure of the LET group and the ABX_LET group exhibited discernible differences, indicating that pseudo germ-free treatment caused a significant influence on the gut flora structure of letrozole-induced mice. Notably, along the PC2 axis, there was a clear distinction of gut microbiota between the ABX_LET group and the ABX group, which accounted for 14.95% of the total microbiota variance (**Figures 4A, B**). These findings suggested that both antibiotic intervention and letrozole modeling independently contributed to changes in the composition of gut flora structure of mice. A random forest model was established on account of the gut microbiota data obtained from four groups of mice through machine learning methods to further analyze the ability of ASVs for the purpose of recognizing different groups. According to the Gini index ranking in the random forest model, when the model error rate was the lowest, Mean Decrease Gini value exceeding 0.1 was selected. A total of 43 key ASVs contributing to the differentiation of Control, LET, ABX, and ABX_LET group were found (**Figure 4C**). Among them, 27 ASVs may contribute to the distinction between Control and LET group, while 29 ASVs may contribute to the distinction between LET and ABX_LET group. And 8 ASVs may contribute to the distinction between ABX and ABX_LET group (**Figure 4C**). The LET group exhibited a significant increase in the absolute abundance of five ASVs compared to the Control group, including bacteria belonging to the unclassified Clostridia_vadinBB60_group, Enterorhabdus, and Muribaculaceae (**Figure 4D**). The absolute abundance of these ASVs was in positive correlation with the TT levels and the area under the IPGTT curve. In addition, the absolute abundance of two ASVs was significantly decreased in LET group, and these microorganisms belonged to the unclassified Clostridia_vadinBB60_group and Enterococcus. The absolute abundance of these two ASVs was significantly negatively correlated with the total testosterone level. Compared with LET group, ABX_LET group significantly reduced the absolute abundance of 5 ASVs, which were ASV62 (unclassified Clostridia_vadinBB60_group), ASV69 (unclassified Enterorhabdus), ASV91 (unclassified Enterorhabdus), ASV51 (unclassified Muribaculaceae), and ASV 52 (unclassified Muribaculaceae). Furthermore, these ASVs with decreased abundance were positively correlated with mice serum total testosterone levels and area under the IPGTT curve (**Figure 4D**).

**Figure 5:**
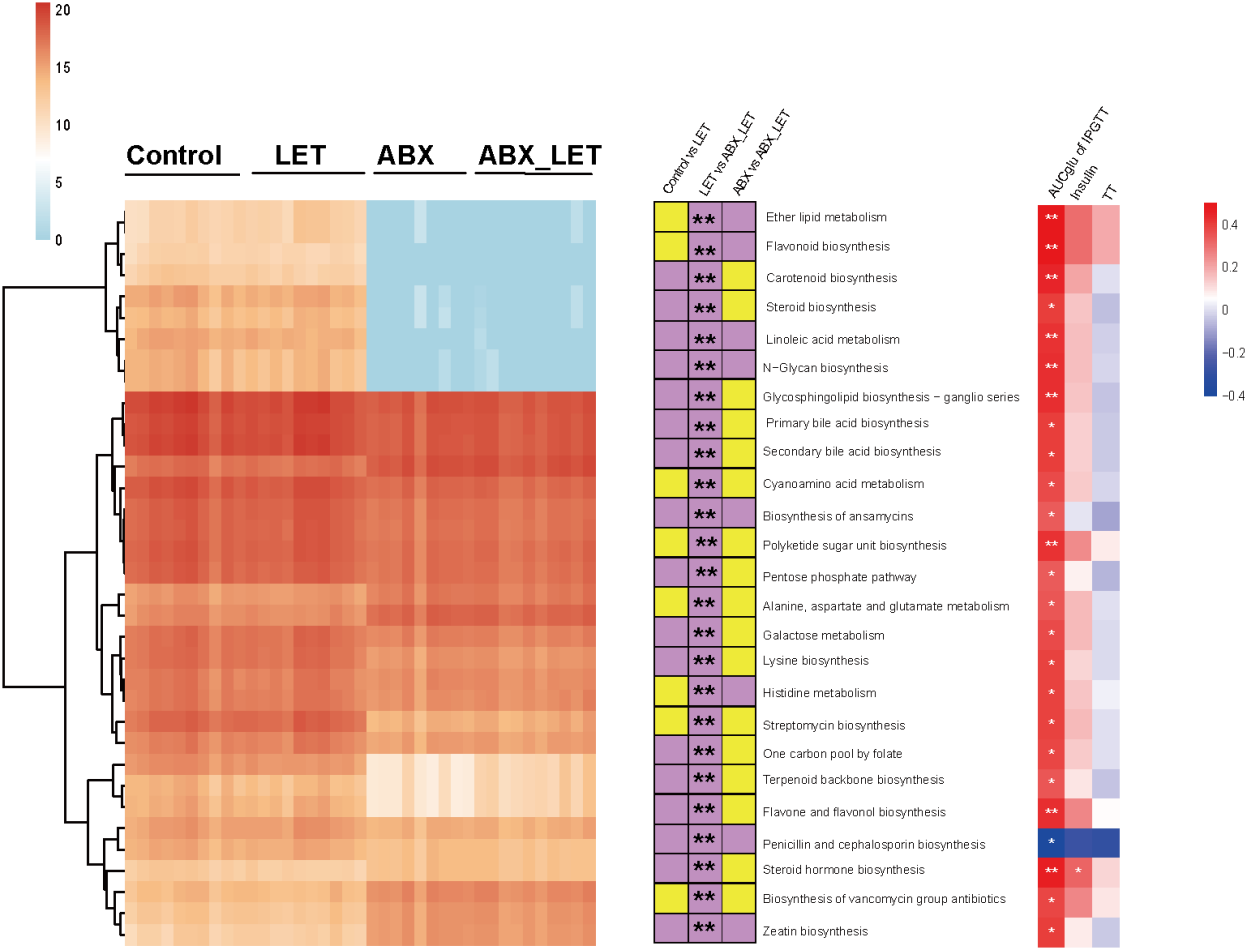
Functional prediction of gut microbiota PICRUSt2. Left: The heat map shows the absolute abundance (transformed logarithmically) of 25 metabolic pathways in each sample. The clustering method is ward. D; In: The stacked bar graph represents the change direction of the absolute abundance of metabolic pathways between groups. If the absolute abundance of metabolic pathways is high (low) in the former, it is purple (yellow). The Mann-Whitney test was used for the difference between groups, *: P<0.05, **: P< 0.01; Right: Spearman correlation analysis of absolute abundance of metabolic pathway with total testosterone, fasting insulin, luteinizing hormone and area under IPGTT curve. Red was positively correlated and blue was negatively correlated, *: P<0.05.

For the purpose of forecasting and comparing the gut flora function of mice in different groups, utilization of PICRUSt2 was implemented based on KEGG database. A total of 25 metabolic pathways displayed significant differences between the LET group and the ABX_LET group, among which the metabolic pathways in the LET group were all elevated compared to those in the ABX_LET group, including the polysaccharide biosynthetic metabolic pathway, lysine synthesis pathway, insulin signaling pathway, linoleic acid metabolism, ether ester metabolism, primary and secondary bile acid synthesis pathway, steroid synthesis pathway, etc. (**Figure 5**). The absolute abundance of these pathways was positively correlated with the area under the IPGTT curve and insulin level (**Figure 5**).

## 4 Discussion

In this study, we preliminarily verified that combined antibiotic intervention by means of eliminating gut microbiota could reduce serum total testosterone level and glucose metabolism disorders in letrozole-induced mice. Nonetheless, the outcomes of the study indicated that there existed no marked amelioration in the level of serum LH and polycystic ovary change. Therefore, it can be inferred that the intestinal flora may not have a crucial impact on the structure of the ovary and LH alterations induced by letrozole modeling.

In a manner akin to the metabolic enhancement observed in the present study, Antibiotic treatment also improved the presence of systemic glucose intolerance, hyperinsulinemia, and insulin resistance in diet-induced obesity subjects by enhancing the secretion of GLP-1 (38, 39). Zarrinpar et al. (25) reported that the elimination of microbiota generated by antibiotics resulted in a reduction of Firmicutes and Bacteroides, as well as noteworthy alterations in SCFA and BA. This phenomenon impacted the gene expression of the intestinal cecum and the signaling of intestinal glucagon-like peptide 1 (GLP-1), facilitated the utilization of glucose by intestinal cells, and consequently led to an amelioration of blood glucose levels, insulin sensitivity, and liver gluconeogenesis. Additional research has corroborated the finding that mice with diet-induced obesity, subjected to high salt and antibiotic-induced microbiome depletion for a duration of 10 weeks, did not exhibit obesity (40). Further investigations into the underlying mechanisms revealed that the reduction of intestinal microbes following ABX treatment can enhance uptake of glucose in brown adipose tissue (BAT) and cecum (41). Ablation of gut microbiota was also certificate to alleviate hyperhomocysteinemia and glucose intolerance in mice induced by a high-methionine diet (42). However, such reduction of microbiota can also impede the heat production competence of BAT through inhibiting the expression of uncoupled protein 1 (UCP1) and the browning procedure of white adipose tissue (43). Nonetheless, there exists heterogeneity among studies, as evidenced by the varying results reported. Specifically, researchers observed that DHEA-induced PCOS mice with depleted gut microbiota exhibited improved hyperinsulinemia and glucose metabolism disorder, but worsened hyperandrogenemia and lipid metabolism disorder (44). This difference may be accounted for discrepancy in antibiotic administration and distinctive modeling techniques. Moreover, Han et al. (6)demonstrated that clearance of intestinal microbiota using ABX reduced serum testosterone levels and free androgen index in the DHEA-treated group, while no significant differences were observed in blood glucose levels.

By using a random forest model, we found 3 groups of gut microbiota were significantly changed after letrozole induction and antibiotic intervention, which were Clostridia_vadinBB60_group, Enterorhabdus, and Muribaculaceae. Moreover, their changes were positively correlated with serum total testosterone levels and area under the IPGTT curve, indicating their important role in regulating glucose metabolism and hyperandrogenemia of mice with PCOS. According to genomic analysis, Muribaculaceae exhibits functional distinctiveness from its neighboring families and possesses multiple roles in the degradation of complex carbohydrates (45). Research indicates that the Muribaculaceae family harbors novel intestinal pathogens, which, when enriched by low-dose sodium glucan sulfate, can migrate to the mice pancreas and induce inflammation in the surrounding area, destruction of beta cell, and insulin-dependent diabetes mellitus (46). Nevertheless, the biological function of individual bacteria within the same family remains unexplored due to the challenges associated with their isolation. Enterorhabdus is regarded to have a significant impact on the modulation of bile acids, fatty acids and amino acids within the gut microbiota (47). Furthermore, Enterorhabdus has been found to be notably more prevalent in non-alcoholic steatohepatitis (NASH), and studies have shown that treatment with UDCA can restore the intestinal microbiome and alleviate liver inflammation in NASH mouse models (48). These findings, in conjunction with population studies, suggest that bile acids may serve as a potential link between hyperandrogenemia, dysglycemia, and the intestinal microbiota.

Through the utilization of PICRUSt2 function prediction analysis, it was determined that antibiotic intervention to the intestinal microbiota of mice with PCOS resulted in alterations to various metabolic pathways, including but not limited to polysaccharide synthesis, primary and secondary bile acid production, lysine synthesis, steroid synthesis, ether ester metabolism, and linoleic acid metabolism. Several researches observed that the circulating concentration of lysine and its metabolite (α-aminoadipic acid) was higher in PCOS patients with metabolic syndrome compared to the individuals of control group. This increase was negatively associated with insulin sensitivity and positively related to the homeostasis model insulin resistance index (49). Additionally, a separate study revealed that the differential metabolites of PCOS and the control group were enriched in the ether ester metabolic pathway (50). The elevation of plasma linoleic acid levels in obese patients with PCOS suggested an augmentation of lipolysis, which could potentially be attributed to compromised insulin function in adipose tissue (51). Researches mentioned above provides evidence for the changes of metabolic pathways. Melanie et al. (52) conducted a metabolomics study and observed distinct alterations in the amino acid spectrum of PCOS patients in both fasting and hyperinsulinemic euglycemic clamp states. Specifically, branched-chain amino acids (BCAAs) were markedly elevated and showed a positive correlation with insulin resistance. Similarly, Chang et al. (49) utilized non-targeted metabolomics analysis in plasma to investigate metabolic syndrome in PCOS and non-PCOS women. They found significant changes in 19 classical pathways in PCOS patients, containing lipid, carbohydrate, amino acid, steroid, and vitamin D metabolism. Further quantitative targeting is warranted.

Therefore, our study showed that antibiotic intervention to eliminate gut microbiota in letrozole mice was capable of reducing serum total testosterone levels and improve glucose metabolism status, but did not significantly improve the morphological changes and LH levels of mice with polycystic ovary syndrome. Functional prediction analysis indicated that several metabolic pathways may be altered. Gut flora could potentially contribute to the onset of hyperandrogenism and glucose metabolism, but the specific mechanism is still unclear. Therefore, our study tried to explore the role gut microbiome played in the pathogenesis of PCOS in mice, and found that depletion of gut microbiota alleviated part of metabolic and reproductive traits. We also performed PICRUSt2 function prediction analysis to forecast transform of various metabolic pathways. However, there still existed some limitations. We did not evaluate alterations in adipose tissue and validate the metabolic pathways predicted above by means of metabolomics and specific biomarkers. Evaluation for sex hormone–binding globulin (SHBG), decreased in in women with PCOS, was also unfeasible due to the absence of postnatal SHBG secretion in mice (16). Moreover, there is strong clinical heterogeneity among PCOS patients, and different phenotypes should be distinguished in the study and demonstration of this disease. In the future, we will further conduct multi-omics studies to clarify the exact role played by intestinal microflora, and conduct fecal bacteria transplantation of human samples to understand the involvement of gut microflora in the pathogenesis of diseases. In the future, enterobacteria-based analysis can be used as a potential tool for the treatment and diagnosis of PCOS, and the integration of clinical landmark, microbiome, and metabolomics can further enhance the differentiation.

## Data availability statement

The data presented in the study are deposited in the NCBI Sequence Read Archive database, accession number PRJNA1020997.

## Ethics statement

The animal studies were approved by the ethics committee of Renji Hospital, Shanghai Jiao Tong University School of Medicine. The studies were conducted in accordance with the local legislation and institutional requirements. Written informed consent was obtained from the owners for the participation of their animals in this study.

## Author contributions

YL: Writing – original draft. YCZ: Writing – original draft. DL: Writing – original draft. WenL: Writing – original draft. YZ: Writing – original draft. WeiL: Writing – original draft, Writing – review & editing. CZ: Writing – original draft, Writing – review & editing. TT: Writing – original draft, Writing – review & editing.
