# Supplementary figures and images for: Depletion of gut microbiota influents glucose metabolism and hyperandrogenism traits of mice with PCOS induced by letrozole
Source: Front Endocrinol (Lausanne). 2023 Oct 20;14:1265152. doi: 10.3389/fendo.2023.1265152 (PMC10623308; doi:10.3389/fendo.2023.1265152)

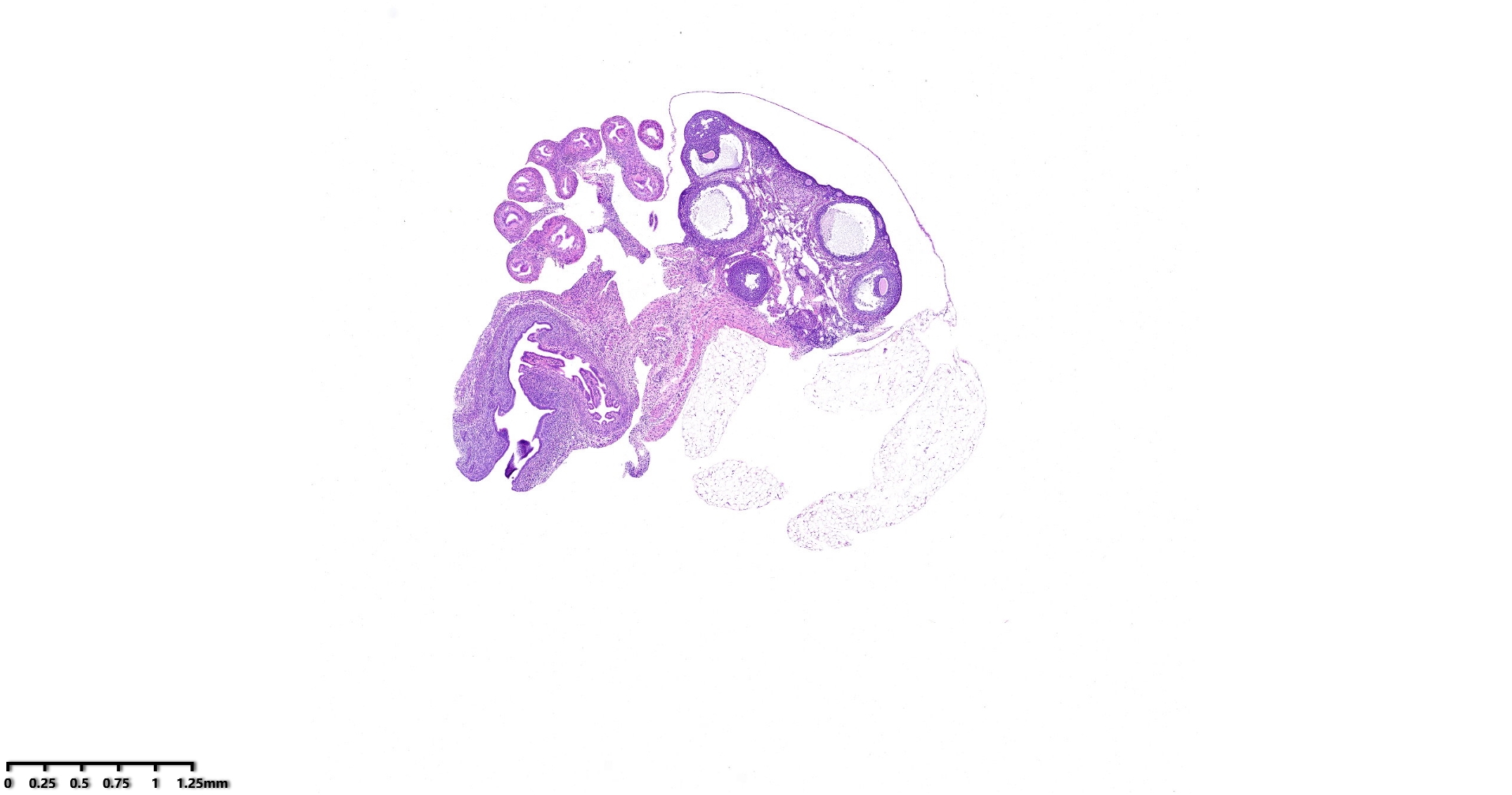

Supplement: Supplementary file 1 [file DataSheet_1.zip › supplementary data/HE/ABX+Letrozole.jpg]

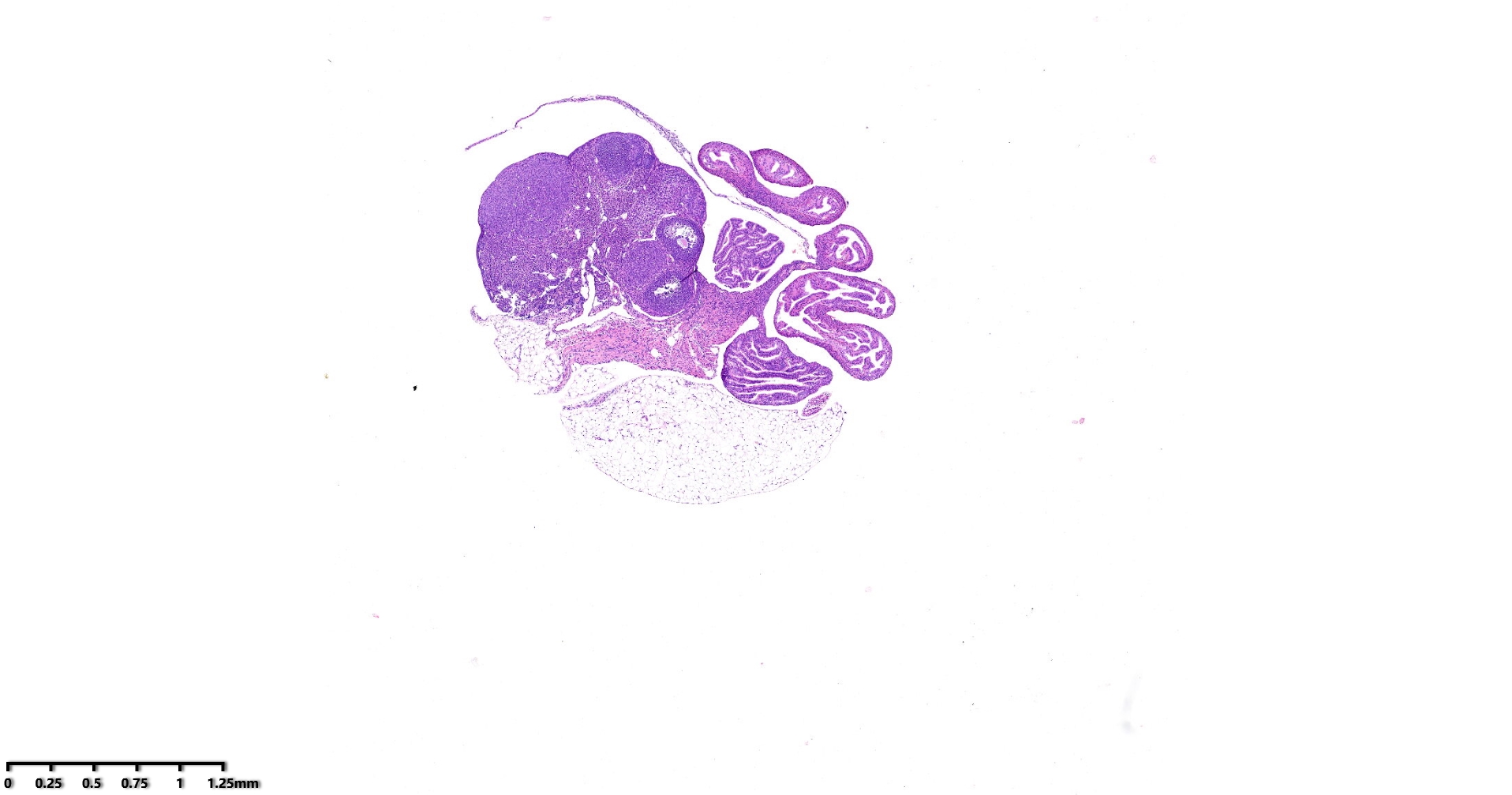

Supplement: Supplementary file 1 [file DataSheet_1.zip › supplementary data/HE/ABX.jpg]

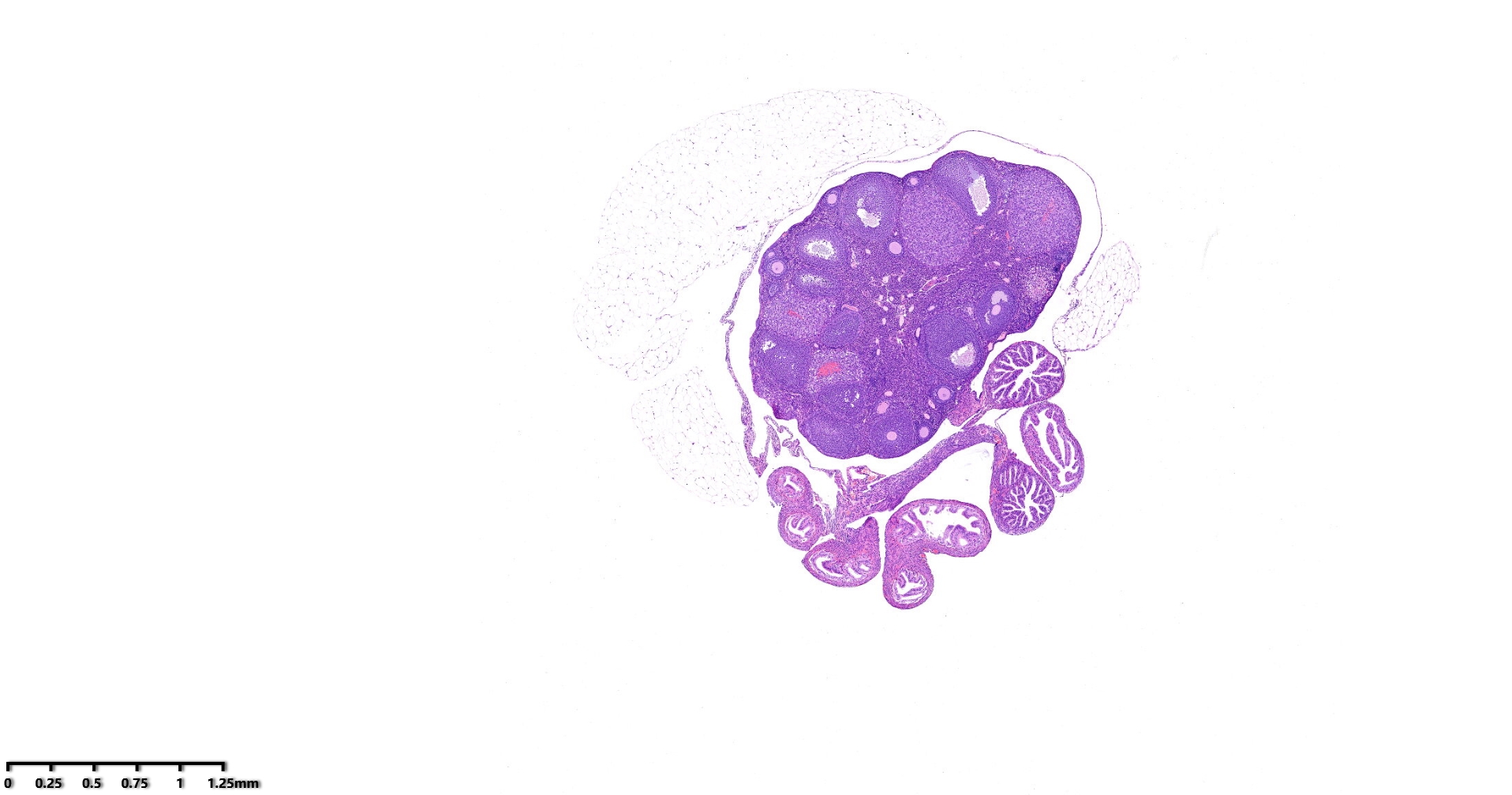

Supplement: Supplementary file 1 [file DataSheet_1.zip › supplementary data/HE/Control.jpg]

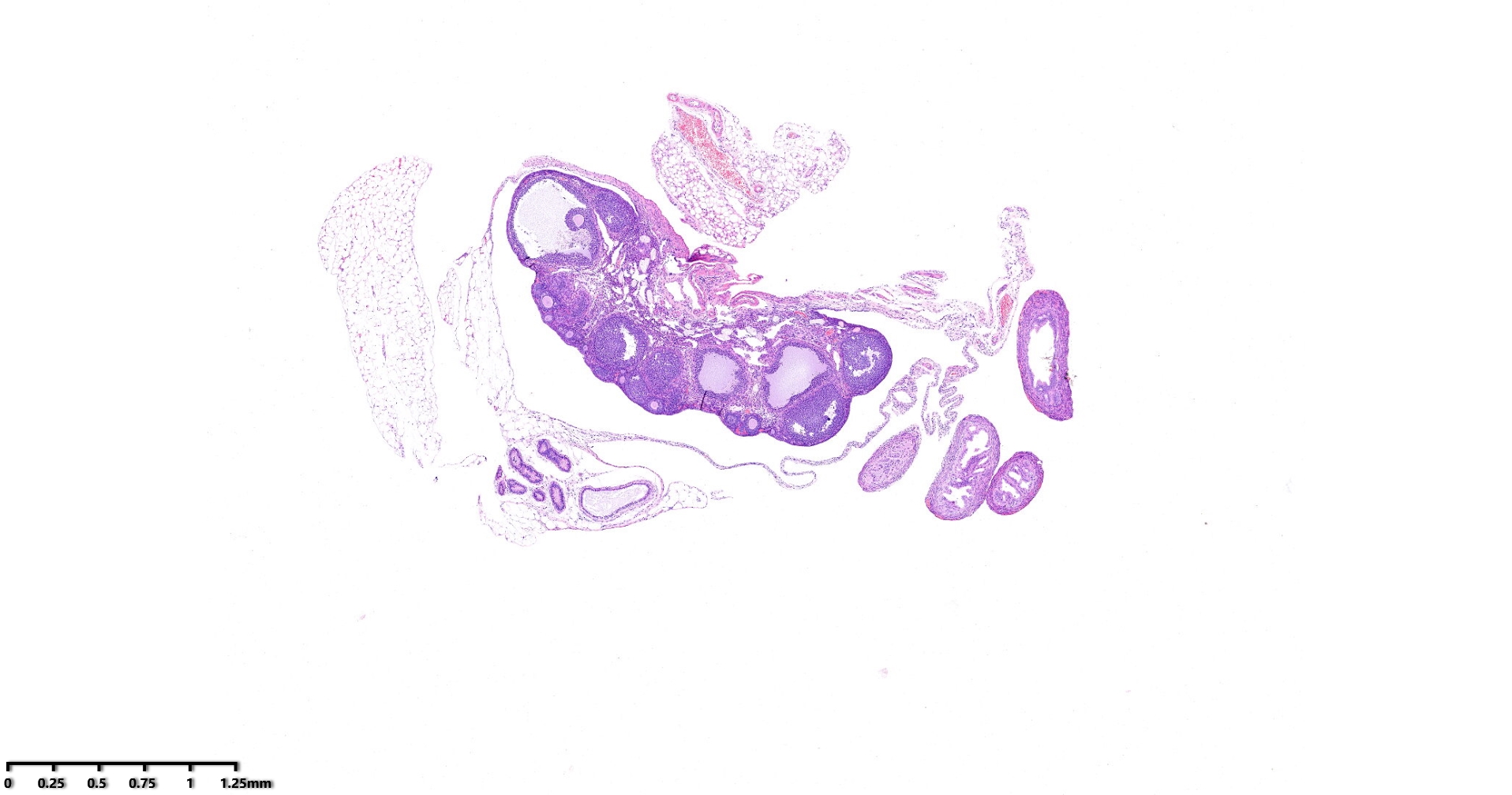

Supplement: Supplementary file 1 [file DataSheet_1.zip › supplementary data/HE/Letrozole.jpg]
